# Supplementary material for: Pulmonary Toxicity of Polystyrene, Polypropylene, and Polyvinyl Chloride Microplastics in Mice
Source: Molecules. 2022 Nov 16;27(22):7926. doi: 10.3390/molecules27227926 (PMC9694469; doi:10.3390/molecules27227926)
Supplement: Supplementary file 1 [file molecules-27-07926-s001.zip › molecules-2009606-supplementary.pdf]

Supplementary Methods

1.1 RNA isolation, library preparation, and sequencing

Total RNA was isolated using Trizol reagent (Invitrogen). RNA quality was assessed using Agilent 2100 bioanalyzer with the RNA 6000 Nano Chip (Agilent Technologies, Amstelveen, Netherlands), and RNA quantification was performed using a ND-2000 Spectrophotometer (Thermo Fisher Scientific). Only samples with an A260/A280 ratio >1.8 and RIN value >7 were considered suitable for use. For control and test RNAs, a library was constructed using QuantSeq 3′ mRNA-Seq Library Prep Kit (Lexogen, Inc., Austria) according to the manufacturer's instructions. Each 500-ng sample of total RNA was prepared and an oligo-dT primer containing an Illumina-compatible sequence at its 5′ end was hybridized, and reverse transcription performed. After degradation of the RNA template, second-strand synthesis was initiated by a random primer containing an Illuminacompatible linker sequence at its 5′ end. The double-stranded library was purified by using magnetic beads to remove all reaction components. The library was amplified to add the complete adapter sequences required for cluster generation. The finished library was purified from PCR components. High-throughput sequencing was performed through single-end 75 sequencing using NextSeq 500 (Illumina, Inc., USA).

1.2 Data analysis

QuantSeq 3′ mRNA-Seq reads were aligned using Bowtie2. Bowtie2 indices were either generated from genome assembly sequences or the representative transcript sequences for aligning to the genome or transcriptome. The alignment file was used for assembling transcripts, estimating their abundances, and detecting differential expression of genes. Differentially expressed genes (DEGs) were determined based on counts from unique and multiple alignments using coverage in Bedtools (Quinlan AR, 2010). Read count (RC) data were processed based on the quantile normalization method using EdgeR within R and Bioconductor. Gene classification was based on searches in the DAVID (http://david.abcc.ncifcrf.gov/).

1.3 Gene ontology (GO) category analysis

To classify the genes altered in PS-instilled C57BL/6 mice with a similar pattern of expression, each gene was assigned to an appropriate category according to its main cellular function. To determine significantly over-represented GO findings, the DAVID functional annotation clustering tool was used by choosing the default option. A Fisher exact test was used to identify significantly enriched pathways and the resulting P values were

adjusted using the BH false discovery rate (FDR) algorithm. Pathway categories with FDR < 0.05 were reported.

Supplementary Table S1. Genes altered in the regulation of eosinophil expression

Symbol	Entrez Gene Name	Fold change	p-value
**CXCL5**	**chemokine (C-X-C motif) ligand 5**	**5.321**	**0.000**
**CCL7**	**chemokine (C-C motif) ligand 7**	**2.183**	**0.041**
**CCL8**	**chemokine (C-X-C motif) ligand 8**	**6.985**	**0.004**
